# Sevoflurane postconditioning attenuates cardiomyocyte hypoxia/reoxygenation injury via restoring mitochondrial morphology

**DOI:** 10.7717/peerj.2659

**Published:** 2016-11-03

**Authors:** Jin Yu, Jianjiang Wu, Peng Xie, Yiliyaer Maimaitili, Jiang Wang, Zhengyuan Xia, Feng Gao, Xing Zhang, Hong Zheng

**Affiliations:** 1 Department of Anethesiology, The First Affiliated Hospital of Xinjiang Medical University, Urumqi, Xinjiang, China; 2 Department of Anethesiology, University of Hong Kong, Hongkong, China; 3 Department of Aerospace Medicine, School of Basic Medical Sciences, Fourth Military Medical University, Xi’an, Shaanxi, China

**Keywords:** Sevoflurane postconditioning, Mitochondria, Hypoxia/reoxygenation, Mitochondrial fusion and fission, Cardiomyocyte

## Abstract

**Background:**

Anesthetic postconditioning is a cellular protective approach whereby exposure to a volatile anesthetic renders a tissue more resistant to subsequent ischemic/reperfusion event. Sevoflurane postconditioning (SPostC) has been shown to exert cardioprotection against ischemia/reperfusion injury, but the underlying mechanism is unclear. We hypothesized that SPostC protects cardiomyocytes against hypoxia/reoxygenation (H/R) injury by maintaining/restoring mitochondrial morphological integrity, a critical determinant of cell fate.

**Methods:**

Primary cultures of neonatal rat cardiomyocytes (NCMs) were subjected to H/R injury (3 h of hypoxia followed by 3 h reoxygenation). Intervention with SPostC (2.4% sevoflurane) was administered for 15 min upon the onset of reoxygenation. Cell viability, Lactate dehydrogenase (LDH) level, cell death, mitochondrial morphology, mitochondrial membrane potential and mitochondrial permeability transition pore (mPTP) opening were assessed after intervention. Mitochondrial fusion and fission regulating proteins (Drp1, Fis1, Mfn1, Mfn2 and Opa1) were assessed by immunofluorescence staining and western blotting was performed to determine the level of protein expression.

**Results:**

Cardiomyocyte H/R injury resulted in significant increases in LDH release and cell death that were concomitant with reduced cell viability and reduced mitochondrial interconnectivity (mean area/perimeter ratio) and mitochondrial elongation, and with reduced mitochondrial membrane potential and increased mPTP opening. All the above changes were significantly attenuated by SPostC. Furthermore, H/R resulted in significant reductions in mitochondrial fusion proteins Mfn1, Mfn2 and Opa1 and significant enhancement of fission proteins Drp1 and Fis1. SPostC significantly enhanced Mfn2 and Opa1 and reduced Drp1, without significant impact on Mfn1 and Fis1.

**Conclusions:**

Sevoflurane postconditioning attenuates cardiomyocytes hypoxia/reoxygenation injury (HRI) by restoring mitochondrial fusion/fission balance and morphology.

## Introduction

Ischemic heart disease remains a major cause of mortality and morbidity worldwide. A rapid recovery of blood flow and oxygen supply after ischemia is a commonly used treatment for myocardial ischemia, but it can result in ischemia-reperfusion injury (IRI) (Zhang & Ren, 2014). To improve clinical outcome in patients suffering from myocardial IRI, innovative treatment strategies to alleviate IRI are needed.

In addition to being an important anesthetic having benefits of limited biotransformation potential and quick patient recovery, sevoflurane can be used as an important cardioprotective agent. Both sevoflurane preconditioning (SPreC) and sevoflurane postconditioning (SPostC) have been shown to have cardioprotective effects similar to that conferred by ischemic preconditioning and postconditioning (Li et al., 2008; Qiao et al., 2013). SPostC is more practical than SPreC in clinical applications (Kloner & Rezkalla, 2006), given that episodes of ischemia are usually unpredictable. Previous studies have indicated multiple mechanisms involved in the cardioprotective effect of SPostC, such as activation of phosphoinositide 3-kinases (PI3K)/protein kinase b (Akt) pathway, upregulation of extracellular signal-regulated kinase 1/2, expressions of hypoxia inducible factor-1alpha and hemeoxygenase-1, prevention of oxidative stress, reductions of reactive oxygen species, rescuing autophagic clearance, and modulation of the activity of apoptotic pathways, with the attenuation of IRI-induced mitochondrial functional impairment as the convergence of various pro-survival signaling pathways (Gao et al., 2016; Li et al., 2008; Yao et al., 2010b; Ye et al., 2012; Yu et al., 2010; Yu et al., 2015). However, the potential underlying molecular mechanism whereby SPostC may attenuate post-ischemic/post-hypoxic myocardial mitochondrial injury and subsequently reduce IRI is still not fully understood.

As the oxygen-consuming power plants for cells, mitochondria provide a critical platform for the synthesis of many essential molecules and allow for highly efficient energy production, through oxidative phosphorylation, which is particularly important in ventricular cardiomyocytes. Mitochondria form a dynamic network constantly changing their morphology by fission and fusion to fulfill the functional needs of the cardiomyocytes. Studies have demonstrated that increased mitochondrial fragmentation (fission) after IRI leads to cell death and tissue necrosis (Givvimani et al., 2015; Ong et al., 2010). Therefore, changes of mitochondrial morphology appear to affect IRI processes and may be fundamental to cardio-protection. However, it is unknown whether SPostC may affect IRI-induced mitochondrial morphology by modulating fission/fusion regulating proteins. This study was designed to investigate how SPostC protects cardiomyocytes against ischemia/reperfusion injury by modulating mitochondrial morphology and affecting mitochondrial fusion- and fission-related proteins in primarily cultured rat cardiomyocytes subjected to hypoxia-reoxygenation.

## Materials and Methods

### Antibodies and reagents

Dulbecco’s modified Eagle’s medium (DMEM), fetal bovine serum and trypsin were purchased from Hyclone (Catalog Number: SH30021.01B, SH30071.03, SH30042.01; Hyclone, Beijing, China). Protease inhibitor, penicillin/streptomycin, Triton X-100 and trypan blue were purchased from Sigma (Catalog Number: S8820, V900929, 72-57-1; Sigma, St. Louis, MO, USA). Phosphatase inhibitor and bovine serum albumin (BSA) were purchased from Merck (Rahway, NJ, USA, Catalog Number: 396073-89-5, 810784). Terminal deoxynucleotidyl transferase-mediated dUTP-biotin nick end labelling (TUNEL) assay was performed using an In Situ Cell Death Detection Kit (POD) from Roche (Catalog Number: 11684817910; Roche, Mannheim, Germany). Lactate dehydrogenase (LDH) kits were purchased from Nanjing Jiancheng Biotech Co. Ltd (Catalog Number: A020-2). Sevoflurane was purchased from Baxter (Rico, Catalog Number: A005A328; Baxter, Guayama, Puerto Rico).

Mitochondrion-Selective Probes MitoTracker® Green FM was purchased from Invitrogen (Catalog Number: M7514; Invitrogen, Foster, CA, USA). Tetramethylrhodamine methylester (TMRM) was purchased from Molecular Probes (Catalog Number: 91055; Molecular Probes, Eugene, CA, USA). The living cell mitochondrial permeability transition pore (mPTP) fluorescence detection kit was purchased from GenMed (GMS10095.1; GenMed, Shanghai).

Rabbit polyclonal antibodies against Mfn1 and Opa1, mouse monoclonal antibodies against Mfn2 and Drp1, horseradish peroxidase (HRP)-conjugated goat anti-rabbit immunoglobulin G (IgG) and anti-mouse IgG were purchased from Abcam (ab104274, ab101589, ab56889, ab56788, ab6721, ab6789; Abcam, Cambridge, MA, USA). A rabbit polyclonal antibody against Fis1 was purchased from Sigma (SAB2702049; Sigma, St. Louis, MO, USA).

### Animals

Clean Sprague-Dawley (SD) neonatal rats (1–3 days old) were purchased from the Experimental Animal Center of The First Affiliated Hospital of Xinjiang Medical University. This study was performed in accordance with the recommendations of the Guide for the Care and Use of Laboratory Animals of the National Institutes of Health. The committee on ethics of animal experiments of the First Affiliated Hospital of Xinjiang Medical University approved all protocols (Permit Number: IACUC-20150225-31). Efforts were made to minimize animal suffering.

### Primary culture of neonatal rat cardiomyocytes (NCMs)

Primary cultures of NCMs were performed as described previously (Tao et al., 2015) with modification. Neonatal rats (1- to 3-day-old SD rats) were sterilized and fixed on a sterilized table top on a clean bench for thoracotomy. Hearts were harvested while beating and washed with a pre-cooled Phosphate buffer saline (PBS) solution at 4 °C until the discarded solution was clear. The vessels, pericardium and atria were carefully removed, and the hearts were cut into l mm^3^ blocks and washed three times with a pre-cooled PBS solution. Heart tissue was transferred to a container and digested with 3–4 cycles with 0.125% trypsin and 0.1% type I collagenase for 8 min at 37 °C under continuous shaking (80 rpm). Cell suspensions were collected in one tube, followed by the addition of DMEM containing 10% fetal calf serum to neutralize the trypsin and filtered through a 200 mesh filter. The collected supernatants were centrifuged at 1,000 rpm for 5 min and maintained in DMEM containing 10% Fetal bovine serum (FBS). Cells were seeded into clean 10 mm dishes and incubated with 5% CO_2_ at 37 °C for 2 h. Purified NCMs were obtained using differential adhesion separation strategies, and cell concentrations were adjusted to 1 × 10^5^ cells/mL before seeding in new dishes. Purified cardiomyocytes were placed in an incubator with 5% CO_2_ at 37 °C, and the medium was changed every 24 h. BrdU (5-bromodeoxyuridine) was added to a final concentration of 0.1 mmol/L to inhibit fibroblast proliferation. The cell culture was incubated for 24 h before experiments were performed to avoid possible contamination from aldosterone or other neurohumoral factors in the serum.

### Experimental protocol

**Figure 1:**
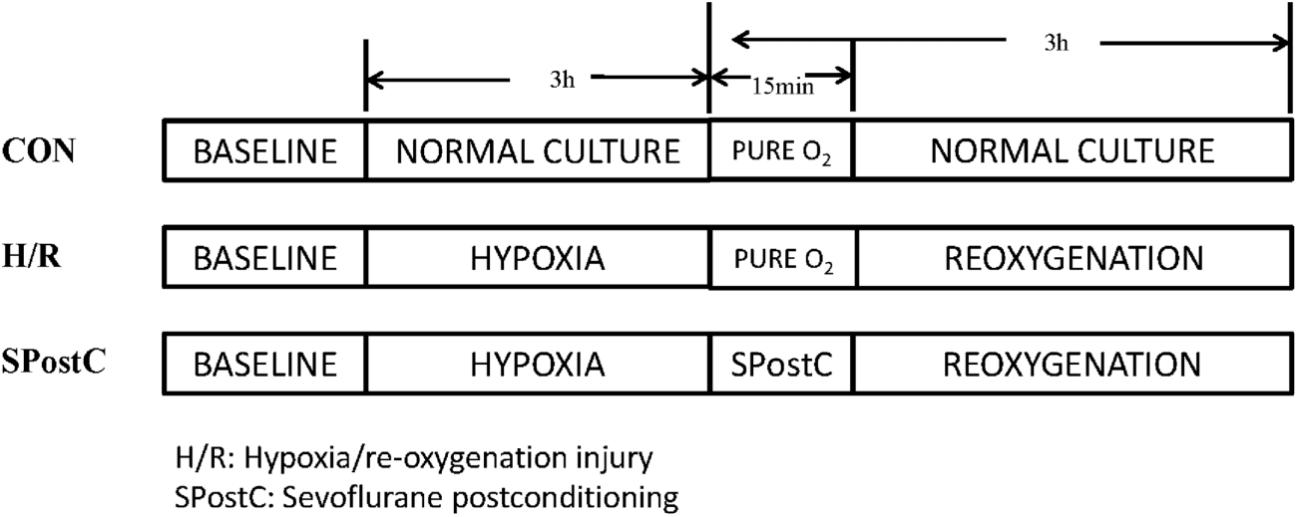
Experimental groups with respective protocols. Cardiomyocytes were randomly divided into control (CON), hypoxia/reoxygenation (H/R) and sevoflurane postconditioning (SPostC) groups.

Cardiomyocytes were randomly divided into three groups (Fig. 1). (1) Control (CON) group: cardiomyocytes were continuously cultured in DMEM medium containing 10% FBS without any interventions but with inhalation of pure oxygen (100% oxygen) for 15 min at the same time when SpostC group was exposed to sevoflurane. (2) Hypoxia/reoxygenation (H/R) group: Cardiomyocytes were incubated in low glucose concentration medium for 48 h, removed, washed with PBS three times, and placed in an airtight container with 95% N_2_ and 5% CO_2_ for 3 h, followed by inhalation of pure oxygen (100% oxygen) for 15 min and then reoxygenation (95% air and 5% CO_2_) with the addition of fresh low glucose DMEM with 10% FBS at 37 °C, for a total 3 h of reoxygenation. (3) SPostC group: Cardiomyocytes were exposed to 2.4% sevoflurane for 15 min at the beginning of reoxygenation after incubation in medium for 48 and 3 h of hypoxia.

### H/R injury of cardiomyocytes

Plates seeded with cardiomyocytes were placed in airtight, humidified, and specifically modified chambers (Modular Incubator Chamber, MIC1; Billups-Rothenberg, Inc., Del Mar, CA, USA; http://www.brincubator.com/) filled with 95% N_2_ and 5% CO_2_ to achieve an oxygen-deficient environment. Ventilation at 5 L/min for 15 min was used to achieve a 1% lower oxygen concentration in the chamber. Cells were incubated at 37 °C for 3 h, and the PBS was removed and replaced with fresh medium containing 10% FBS. Longer 3 h incubation in 95% air and 5% CO_2_ at 37 °C was performed as reoxygenation. The plates of CON group were kept in normoxic conditions for the corresponding times.

### Post-hypoxic Sevoflurane Postconditioning (SPostC) of cardiomyocytes

A Vapor 2000 sevoflurane vaporizer (Drager, Germany) was used to apply a gas mixture containing 97.6% O_2_ and 2.4% sevoflurane (Sevoflurane, Baxter, A005A328, Guayama). Cells were exposed to sevoflurane as previously described (Liu et al., 2015a). Briefly, an in-line sevoflurane vaporizer fed a supply of gas mixture containing 97.6% O_2_ and 2.4% sevoflurane for at least 10 min until the desired sevoflurane concentration (2.4%) was achieved. Concentrations of sevoflurane and O_2_ were monitored using an anesthetic analyzer (Drager Vamous, Germany) in the outlet. The gas flow rate was 2 L/min. Cells were transferred to the incubator after exposure. The sevoflurane concentration was chosen according to a previous study, which determined that this concentration in vivo resulted in optimal cardioprotective effects in rats (Obal et al., 2001). Other groups that do not require the intervention of sevoflurane received inhalation of pure oxygen for the same time interval.

### Lactate dehydrogenase detection

Supernatant (0.1 ml) was collected from each group 1 min after reoxygenation to detect LDH activity using LDH kits according to the manufacturer’s instructions (Nanjing Jiancheng Biotech Co. Ltd) as we described (Xu et al., 2013). LDH activity was expressed as international units per liter (IU/L). N = 6 from three independent experiments.

### Assessment of apoptotic cell death

TUNEL (TdT-mediated dUTP nick-end labelling) was used for the detection of cell death following the protocol from the Roche POD, and the results were analyzed using fluorescence microscopy (Olympus DP72, Japan) and Image Pro Plus software (Media Cybernetics, MD, USA) as described (Liu et al., 2015b). Thirty visual fields were randomly selected for calculations of the average result from three repeated independent experiments. At least 30 randomly chosen visual fields with 600–800 cells were scored, and the number of TUNEL-positive cells was expressed as a fraction of the total cell number.

### Cardiomyocyte viability

Cardiomyocyte viability was analyzed using a trypan blue exclusion (TBE) assay. Briefly, nine drops of cell suspension were placed in a test tube and one drop of a 0.4% trypan blue (Sigma, CAS NO72-57-1) solution was added and mixed. Living and dead cells were counted rapidly in 3 min. Thirty visual fields under a 20× objective lens were randomly selected from three independent experiments for quantification, and an average result was obtained. The percentage of cell survival and viability was calculated as: non-TBEs/(non-TBEs + TBEs)×100%.

### Mitochondrial morphology analysis

To observe the morphological changes of mitochondria, an Image J macro was used to measure mitochondrial interconnectivity and elongation from confocal fluorescence micrographs of cells. Errors were minimized by selecting enough numbers of cells from independent experiments and calculating from two-dimensional planar photographs. Mitochondrial morphology in cardiomyocytes was captured using a confocal microscope (Olympus, FV10-ASW). A final concentration of 200 nm Mito-Tracker Mitochondrion-Selective Probes (Invitrogen, MitoTracker® Green FM, M7514) was incubated with cardiomyocytes for 30 min. Samples were excited at 489 nm, and emission at 510 nm from a laser source was measured. Thirty randomly chosen cardiomyocytes per treatment group (n = 3 independent experiments with 10 incubated cardiomyocytes per experiment) were analyzed. An Image J macro (publicly available for download from the official ImageJ site: https://imagej.nih.gov/ij/) was created to quantify two parameters of mitochondrial morphology. The green channel of cardiomyocytes stained with MitoTracker Green FM (200 nm; Molecular Probes) was extracted to grey scale, inverted to show mitochondria-specific fluorescence as black pixels and set to optimally resolve individual mitochondria. The macro traced mitochondrial outlines using ‘analyse particles.’ The mean area/perimeter ratio was used as an index of mitochondrial interconnectivity. The inverse circularity was used as a measure of mitochondrial elongation. These indices were validated as good characterized mediators of mitochondrial fission and fusion (Dagda et al., 2009).

### Mitochondrial membrane potential measurement

TMRM is used in the ‘redistribution mode’ to assess mitochondrial membrane potential (Δψm), and therefore, a reduction in mitochondrial localization in TMRM fluorescence represents mitochondrial depolarization. Mitochondrial membrane potential was measured using TMRM according to the manufacturer’s instructions. Cells (in medium) were incubated with 20 nmol/L TMRM and monitored at 544/574 nm using fluorescence microscopy after reoxygenation. Fluorescence intensities were analyzed using Image J software. Twenty images were randomly selected for each treatment group from three independent experiments.

### Detection of mPTP opening

mPTP opening detection method in intact cells was established in previous studies (Saotome et al., 2009). Cardiomyocytes were plated in confocal dishes according to the manufacturer’s instructions to measure mPTP opening in intact cells. The cardiomyocyte culture medium was removed, and the cells were treated with 1 μM Calcein-AM + 1 mM CoCl_2_ Calcein-AM, which is capable of entering the cytosol and mitochondria. Calcein was entrapped via de-esterification at 37 °C. The extent of mPTP opening was measured by the loss of mitochondrial calcein fluorescence. Fluorescence intensities were measured using ImageJ software. Six or seven images were randomly selected for each treatment group and repeated in three independent experiments for a total of 20 images per treatment group.

### Immunofluorescence staining

After reperfusion, cardiomyocytes were washed with PBS three times and were fixed in 4% paraformaldehyde at room temperature for 15 min. They were then permeabilized in 0.1% Triton X-100 for 20 min and incubated either overnight at 4 °C or 2 h at 21 °C (depending on the primary antibody) with primary antibodies for Mfn1 and Opa1 at a dilution of 1:200 and Mfn2, Drp1 and Fis1 at 1:400 in PBS. Samples were washed three times with PBS and incubated at 37 °C for 40 min with anti-mouse or anti-rabbit second antibodies (1:400 dilutions; Santa Cruz Biotechnology). Washed cardiomyocytes were incubated for 15 min at 37 °C with 4′,6-Diamidino-2-Phenylindole, Dihydrochloride (DAPI) at a dilution of 1:1,000. Samples were washed again with PBS and mounted on microscopic cover slip. Images were captured using confocal microscopy for visualization of related proteins (described above) and DAPI. Fluorescence intensities were analyzed using ImageJ software. Thirty treated cells were randomly selected for analysis from each treatment group.

### Western blotting

NCMs were kept on ice after reoxygenation and washed twice with PBS. Proteins were solubilized and extracted with 100 μL of RIPA buffer (Radio-Immunoprecipitation Assay (RIPA)/Phenylmethanesulfonyl fluoride (PMSF): 100/1) for each dish, and the lysates were used to estimate protein content using Bradford Assay Reagent. Samples were boiled for 5 min at 95 °C in 5× loading buffer, and equivalent amounts of protein preparations (20 μg) were separated on 10% SDS-polyacrylamide gels (SDS-PAGE) in running buffer. Separated proteins were transferred to Polyvinylidene fluoride (PVDF) Western blotting membranes (Roche, UK). Membranes were blocked in 5% non-fat milk for 1 h and probed with appropriate primary antibodies (1:1,000) overnight at 4 °C followed by incubation with peroxidase-conjugated secondary antibodies. Signals were detected using enhanced Pierce chemiluminescence and visualized on X-ray films. Tubulin served as the loading control. Proteins were expressed as ratios normalized to β-tubulin (n = 5/group).

### Statistical analysis

Statistical analysis was performed using GraphPad Prism 6. All values are expressed as the mean ± SEM. Differences between CON and experimental groups were determined using one-way analysis of variance (ANOVA) followed by Tukey’s multiple comparisons test for every two groups. P-values < 0.05 were considered significant.

## Results

### SPostC reduced cell death and LDH level, increased cell viability following simulated H/R

**Figure 2:**
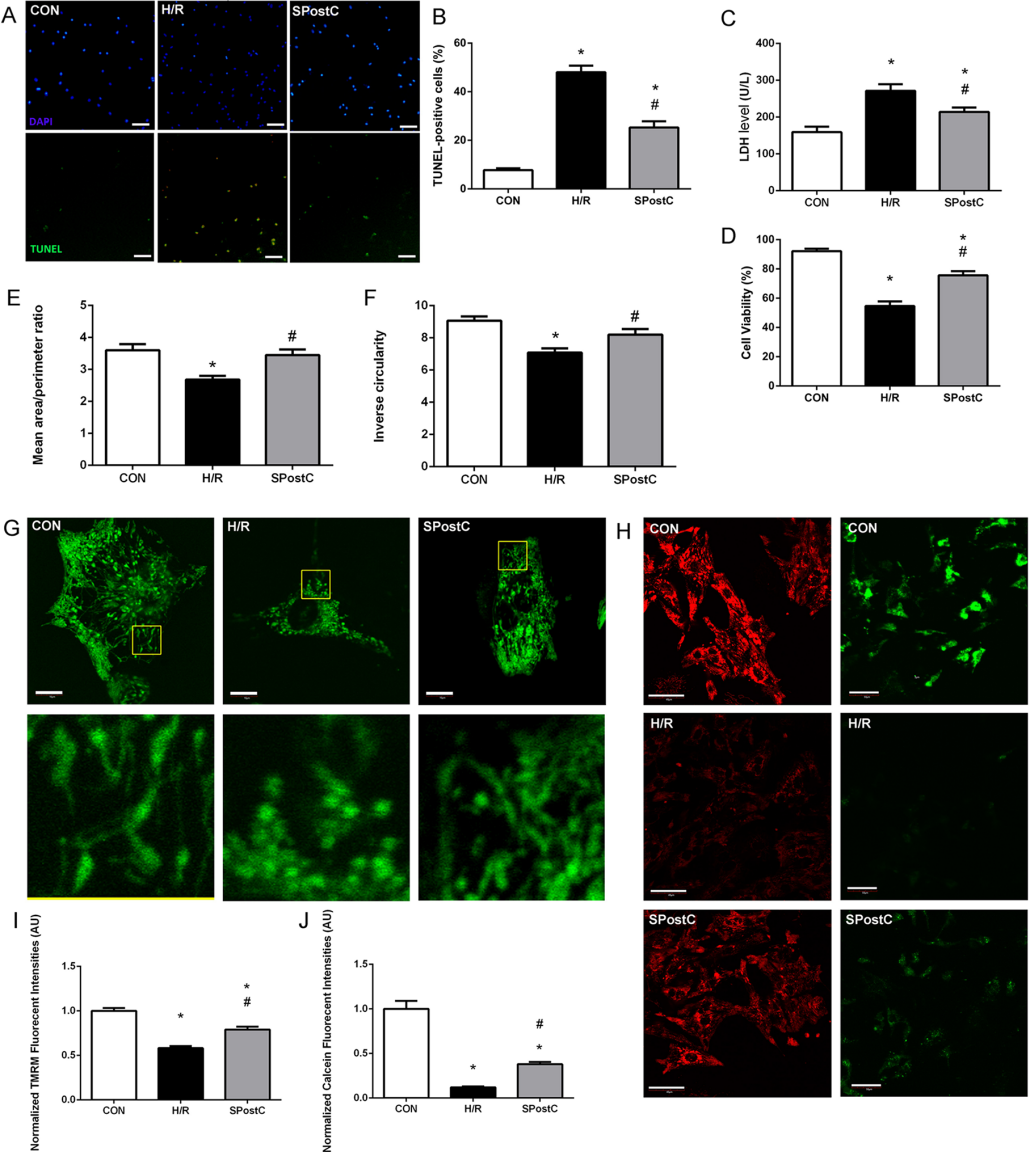
SPostC decreased LDH level, cell death and cell viability, increased mitochondrial interconnectivity, elongation and membrane potential but decreased the opening sensitivity of mPTP on post-hypoxic primary cultured neonatal rat cardiomyocytes (NCMs). (A) Representative images of TUNEL visualizations (10×), Scale bar: 100 μm. (B) SPostC decreased cell death compared with H/R group; data represent mean ± SEM from three independent experiments. (C) SPostC decreased LDH level compared with H/R group, (n = 6 from three independent experiments). (D) SPostC increased cell viability compared with H/R group; data represent mean ± SEM of ten visions from three independent experiments in each group. Quantitative image analysis revealed increased mean area/perimeter ratio (E) and mitochondrial inverse circularity (F) with SPostC (n = 30 cells/per group). (G) Representative images of mitochondrial morphology of three groups (Scale bar: 10 μm). (H) Representative images of TMRM fluorescence (Scale bar: 40 μm, red fluorescence) and Calcein fluorescence (Scale bar: 50 μm, green fluorescence) of three groups. TMRM staining was used in the ‘redistribution mode’ to assess Δψm, and TMRM fluorescence intensities represent the mitochondrial membrane potential (Δψm). mPTP opening was measured by the loss of mitochondrial calcein fluorescence. (I) SPostC increased the normalized TMRM fluorescent intensities (J) comparing with H/R group but prevented the fluorescence decreasing under H/R condition. (n = 20 visions/per group). (*p < 0.05 compared with control group, #p < 0.05 compared with H/R group).

Representative images of TUNEL visions are shown in Fig. 2A, blue dots represent all nuclei, and green dots represent dead cell nuclei. SPostC reduced cell death (25.26 ± 2.58% in SPostC vs 48.04 ± 2.68% in the H/R, p < 0.05; 7.72 ± 0.78% in the CON group) and LDH level (214.33 ± 11.75 IU/L in SPostC vs 271.50 ± 17.76 IU/L in the H/R, p < 0.05; 159.04 ± 14.83 IU/L in CON group) in primary cultured neonatal cardiomyocytes subjected to H/R comparing with untreated H/R group (Figs. 2B and 2C). SPostC also increased the cell viability following hypoxia/reoxygenation injury (HRI) comparing with H/R group (75.61 ± 2.89% in SPostC vs 54.62 ± 3.17% in the H/R, p < 0.05; while 92.15 ± 1.63% in the CON group) (Fig. 2D).

### SPostC modulated mitochondrial morphology, maintained mitochondrial membrane potential and inhibited mPTP opening

As mitochondrial networks are analyzed more effectively by whole cell analysis than by electron microscopy, we utilized computer automated image analysis to explore the morphologic effects of SPostC. The custom macro for NIH ImageJ traces individual mitochondria in an unbiased manner and computes indices of mitochondrial interconnectivity and mitochondrial elongation. Mitochondrial interconnectivity was significantly impaired in primary cultured neonatal cardiomyocytes subjected to H/R, and SPostC treatment altered mitochondrial morphology manifested as increased mitochondrial interconnectivity (mean area/perimeter ratio: 3.45 ± 0.14 in SpostC vs 2.69 ± 0.11 in the H/R, p < 0.05; while the value in CON group was 3.60 ± 0.14) and increased mitochondrial elongation (inverse circularity: 8.20 ± 0.32 in SpostC vs 7.09 ± 0.18 in the H/R, p < 0.05; while the value in CON group was 9.07 ± 0.26) as shown in Figs. 2E and 2F. Representative images of mitochondrial morphology of three groups are shown in Fig. 2G. Representative images of TMRM fluorescence (Red) and Calcein fluorescence (Green) of three groups are shown in Fig. 2H. TMRM fluorescent intensities were detected to measure mitochondrial membrane potential (Δψm) of each experimental group. SPostC increased the normalized TMRM fluorescent intensities compared with H/R group (0.79 ± 0.02 in SPostC vs 0.58 ± 0.01 in H/R, p < 0.05, 1.00 ± 0.02 in CON group, Fig. 2I). Calcein fluorescence was greatly decreased in H/R group compared with CON group, which reflected the increased opening sensitivity of mPTP, and SPostC prevented the fluorescence decreasing under H/R conditions (0.38 ± 0.03 in SPostC vs 0.12 ± 0.01 in H/R, p < 0.05, 1.00 ± 0.09 in CON group) (Fig. 2J).

### Effects of SPostC on H/R induced alterations of mitochondrial fusion and fission proteins

**Figure 3:**
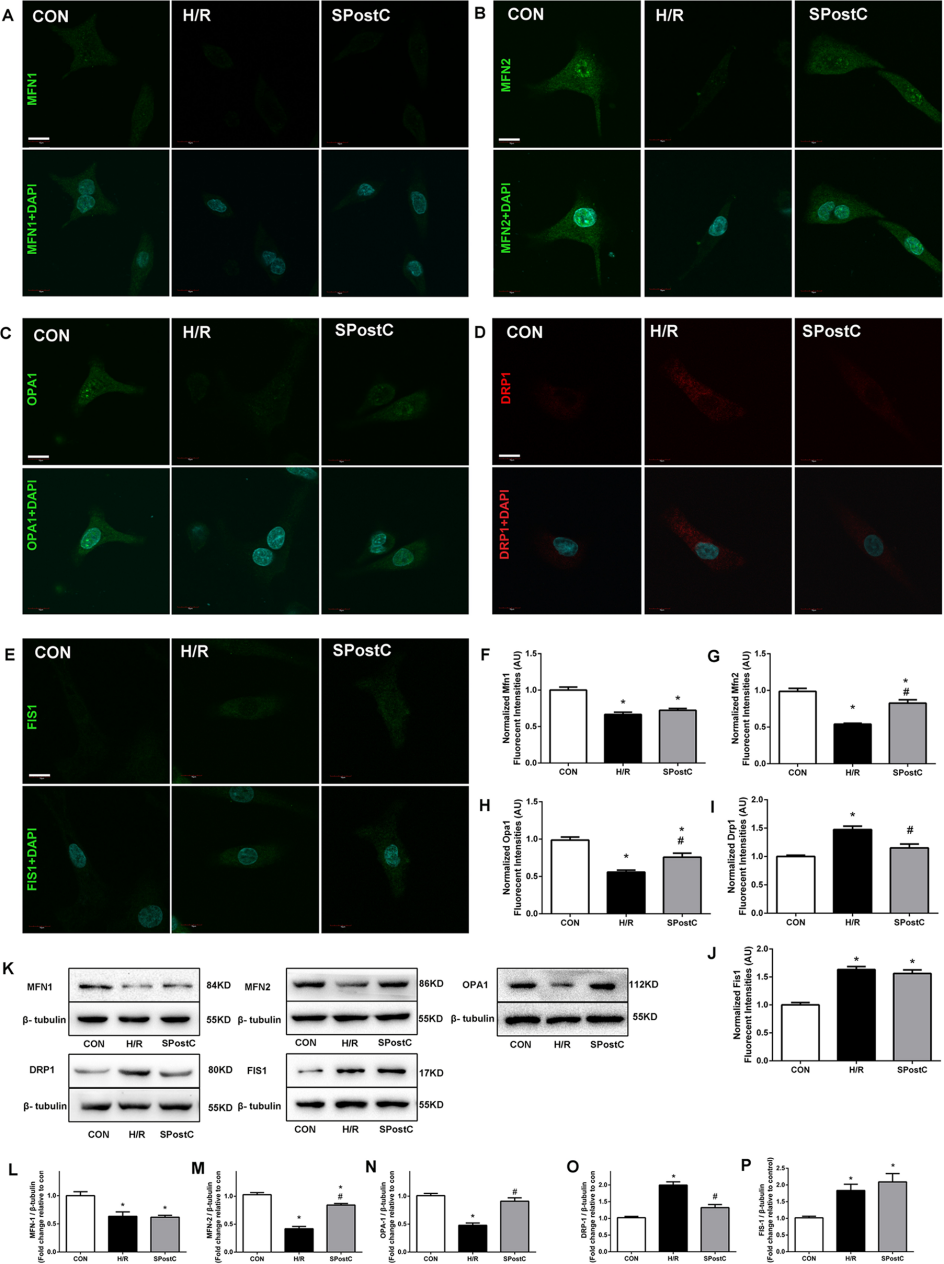
Effect of SPostC on mitochondrial fusion and fission protein expression after H/R injury. (A–E) Mitochondrial fusion proteins (Mfn1, Mfn2 and Opa1) and fission proteins (Drp1 and Fis1) were analyzed using immunofluorescence and western blotting with specific antibodies. Fluorescent intensity in randomly selected cell after reoxygenation was analyzed. N = 30 cells/group, Scale bar: 10 μm. Protein content was normalized to anti-β-tubulin in western blot assay, n = 5. Representative immunofluorescence obtained by confocal microscope of five proteins from three groups. (F) Fusion proteins Mfn1; (G) Fusion proteins Mfn2; (H) Fusion proteins Opa1; (I) Fission protein Drp1; (J) Fission protein Fis1. (K) Representative blots. (L) Fusion proteins Mfn1; (M) Fusion proteins Mfn2; (N) Fusion proteins Opa1; (O) Fission protein Drp1; (P) Fission protein Fis1. Data are the means ± SEM from three separate experiments. (*p < 0.05 vs control group, #p < 0.05 vs H/R group, one-way ANOVA).

Mitochondrial morphology changed with SPostC, as indicated by modulation of mitochondrial fusion and fission protein expression. Representative immunofluorescence obtained by confocal microscope of 5 proteins is shown in Fig. 3A–3E. For Mfn1, SpostC did not increase the expression of Mfn1 compared with H/R group (0.72 ± 0.01 in SpostC vs 0.67 ± 0.01 in H/R, p > 0.05, 1.00 ± 0.02 in CON group, Fig. 3F). SpostC increased the expression of Mfn2 compared with H/R group (0.83 ± 0.02 with SpostC vs 0.54 ± 0.01 in H/R, p < 0.05, 1.00 ± 0.03 in CON group, Fig. 3G). SpostC increased the expression of Opa1 compared with H/R group (0.76 ± 0.04 with SpostC vs 0.56 ± 0.02 in H/R, p < 0.05, and 1.00 ± 0.03 in CON group, Fig. 3H). For fission-related protein Drp1, SpostC decreased the expression of Drp1 compared with H/R group (0.97 ± 0.02 in SpostC vs 1.11 ± 0.02 in H/R, p < 0.05, and 1.00 ± 0.02 in CON group, Fig. 3I), but SpostC did not affect the expression of Fis1 compared with H/R group (1.18 ± 0.02 in SpostC vs 1.12 ± 0.02 in H/R, p > 0.05, and 1.00 ± 0.01 in CON group, Fig. 3J). Representative protein bands obtained by western blot are shown in Fig. 3K, protein content was normalized to anti-β-tubulin in western blot assay, the variation trend of above five proteins were consist with results from immunofluorescence (Fig. 3L–3P).

## Discussion

The present study confirmed that SPostC effectively protected the cardiomyocytes against H/R injury in primary cultured NCMs, as evidenced by reduced LDH level and cell death and increased cell viability following simulated H/R. This finding is in line with the proven SPostC cardiac protective effects reported in related models (Gao et al., 2016). In particular, we found that mitochondrial fusion and fission changes were involved in the potential molecular mechanisms of SPostC, and consequently may be the major mechanism whereby SPostC significantly inhibits mPTP opening and maintains mitochondrial membrane potential. Moreover, SPostC-induced mitochondrial network changes were associated to mitochondrial fusion- and fission-related proteins (Mfn2, Opa1 and Drp1). The influence of hypoxia/ischemic on fusion and fission proteins were in line with vitro studies (Dong et al., 2016; Reshma et al., 2016) on H9C2 cells and vivo study on rats (Yu et al., 2015).

SPostC has been shown to enhance endothelial and cardiac resistance to IRI both in experimental (Li et al., 2015) and in clinical settings (Wang et al., 2013). While some factors have been linked to the protective effects of SPostC, the underlying mitochondrial-related mechanism is not fully understood. Researchers have demonstrated in vivo that pharmacological inhibition of the mitochondrial fission protected cardiomyocytes against IRI by inhibiting mPTP opening and reducing myocardial infarct size in murine heart (Ong et al., 2010). Further research confirmed that Akt protects the heart by modulating mitochondrial morphology (Ong et al., 2015). However, whether SPostC can affect mitochondrial morphology, until now, has not been examined. As SPostC also reduced apoptosis after myocardial IRI via the activation of PI3K/AKT/mTOR signaling (Zhang et al., 2014), we postulated that SPostC protects cardiomyocytes via modulating mitochondrial morphology, and the experimental results confirmed our hypothesis.

Mitochondria have been shown in recent years to be highly dynamic organelles with varied roles in defending against myocardial injury (Din et al., 2013; Disatnik et al., 2013; Gao et al., 2013; Givvimani et al., 2015; Parra et al., 2008). Mitochondrial morphology adjustment modulates the susceptibility of cardiomyocytes to cell injury during acute IRI (Ong et al., 2010). Our study here confirmed the relationship between SPostC and mitochondrial morphology. From the results, we gain a preliminary understanding that the modulation of mitochondrial morphology is involved in the cardioprotection of SPostC by fusion- and fission-related proteins expression. Further laboratory experiments such as genetic level analysis will be carried out to support this conclusion.

SPostC has been confirmed to inhibit opening of mPTP via multiple signaling pathways (He et al., 2008; Yao et al., 2009; Yao et al., 2010a; Yao et al., 2010b; Yao, Liu & Li, 2016). The opening of mPTP at the onset of reperfusion plays a key role during IRI and mediates cell death. In our study, the degree of mPTP opening were corresponded to the extent of mitochondrial morphological changes, which provides evidence to show that mitochondrial function is closely related to its morphology during IRI.

Findings from this study extended the results of a recent study which showed that SPostC protected the rat heart via ameliorating mitochondrial impairment and oxidative stress by rescuing autophagic clearance, suppressing the decline of Opa1, and therefore inhibiting Drp1 and Parkin induced by IRI (Yu et al., 2015). Our results provide evidence to suggest that SPostC may maintain/restore post-hypoxic mitochondrial morphology by modulating mitochondrial fusion- and fission-related proteins.

Of note, SPostC did not affect all of the mitochondrial fusion- and fission-related proteins. SPostC restored the fusion-related proteins expression of Mfn2 and Opa1 but not Mfn1, although Mfn1 and Mfn2 share homology. It seems likely that Mfn1 is not as important in mitochondrial fusion as Mfn2, due to differences in their GTPase activity (Ishihara, Eura & Mihara, 2004). Loss of Mfn2 has induced dysfunction, whereas loss of Mfn1 has been well tolerated (Chen & Dorn, 2013). Mfn2 (but not Mfn1) can localize to the endoplasmic reticulum (and sarcoplasmic reticulum in cardiomyocytes), thus tethering ER/SR to mitochondria and facilitating calcium signaling in the organelle (Chen et al., 2012; de Brito & Scorrano, 2008). Mfn2 can also act as a mitochondrial receptor protein for Parkin translocation during mitophagic organelle culling (Chen & Dorn, 2013). On the other hand, SPostC decreased the fission-related protein expression of Drp1, but not Fis1. Fis1 is a small protein located in the outer mitochondrial membrane to help Drp1 play its role in mitochondrial fission. Other small proteins (e.g., MFF, MiD49, MiD51) can recruit Drp1 independent of Fis1 (Loson et al., 2013). On the basis of this analysis and previous studies, the effect of SPostC on these proteins remains to be further studied. Given the complexity of the mitochondrial fusion and fission regulation process, signaling pathways such as PI3K/AKT or HIF-1α/HO-1 which were not investigated here may be involved in this controversial phenomenon. Thus, further studies to explore the relationship between the mitochondrial morphology changes or mPTP and some signaling pathways should be considered.

## Conclusions

Overall, our data demonstrated that SPostC protects the cardiomyocytes against H/R injury by modulating mitochondrial morphology, inhibiting mPTP opening and maintaining mitochondrial membrane potential. The effects were related to modulation of Mfn2, Opa1 and Drp1 proteins. These findings reveal a new insight into the mechanism of SPostC cardioprotection and open the way for targeted treatment. Further studies are needed to clarify the causal linkage between these mechanisms.

## Supplemental Information

10.7717/peerj.2659/supp-1 Supplemental Information 1 Raw data for all indicators of the three group, CON group, H/R group and SPostC group. Raw data for LDH level, cell death, cell viability, mitochondrial morphology analysis, mitochondrial membrane potential measurement, detection of mPTP opening, and related protein expression result.

10.7717/peerj.2659/supp-2 Supplemental Information 2 Comparison results of all indicators among three group. Comparison results of LDH level, cell death, cell viability, mitochondrial morphology analysis, mitochondrial membrane potential measurement, detection of mPTP opening, and related protein expression. All values are expressed as the mean ± SEM.

10.7717/peerj.2659/supp-3 Supplemental Information 3 Mito-Morphology Macro. Measures the cellular mitochondrial content in each cell and mitochondrial morphology with this Macro.

10.7717/peerj.2659/supp-4 Supplemental Information 4 Full length uncropped blots of five proteins.

## Additional Information and Declarations

### Data Deposition

The following information was supplied regarding data availability: The raw data has been supplied as Supplemental Dataset Files.
