# Supplementary material for: Sevoflurane postconditioning attenuates cardiomyocyte hypoxia/reoxygenation injury via restoring mitochondrial morphology
Source: PeerJ. 2016 Nov 3;4:e2659. doi: 10.7717/peerj.2659 (PMC5101611; doi:10.7717/peerj.2659)
Supplement: Supplemental Information 2 — Comparison results of LDH level, cell death, cell viability, mitochondrial morphology analysis, mitochondrial membrane potential measurement, detection of mPTP opening, and related protein expression. All values are expressed as the mean ± SEM. [file peerj-04-2659-s002.docx]

|  | CON | H/R | SPostC |
| --- | --- | --- | --- |
| LDH level (U/L) | 159.04±14.83 | 271.50±17.76 | 214.33±11.75 |
| Cell death（%） | 7.72±0.78 | 48.04±2.68 | 25.26±2.58 |
| Cell viability（%） | 92.15±1.63 | 54.62±3.17 | 75.61±2.89 |

Data represent mean±SEM.

|  | CON | H/R | SPostC |
| --- | --- | --- | --- |
| Mean area/perimeter ratio | 3.60±0.14 | 2.69±0.11 | 3.45±0.14 |
| Inverse circularity | 9.07±0.26 | 7.09±0.18 | 8.20±0.32 |
| Normalized TMRM fluorescence | 1.00±0.02 | 0.58±0.01 | 0.79±0.02 |
| Normalized Calcein fluorescence | 1.00±0.09 | 0.12±0.01 | 0.38±0.03 |

Data represent mean±SEM.

|  | CON | H/R | SPostC |
| --- | --- | --- | --- |
| Normalized fluorescent intensity of MFN1 | 1.00±0.02 | 0.67±0.01 | 0.72±0.01 |
| Normalized fluorescent intensity of MFN2 | 1.00±0.03 | 0.54±0.01 | 0.83±0.02 |
| Normalized fluorescent intensity of OPA1 | 1.00±0.03 | 0.56±0.02 | 0.76±0.04 |
| Normalized fluorescent intensity of DRP1 | 1.00±0.02 | 1.11±0.02 | 0.97±0.02 |
| Normalized fluorescent intensity of FIS1 | 1.00±0.01 | 1.12±0.02 | 1.18±0.02 |
| MFN1 / β-tubulin  (Fold change relative to control) | 1.00±0.07 | 0.63±0.08 | 0.62±0.03 |
| MFN2 / β-tubulin  (Fold change relative to control) | 1.00±0.04 | 0.42±0.04 | 0.84±0.03 |
| OPA1 / β-tubulin  (Fold change relative to control) | 1.00±0.04 | 0.48±0.04 | 0.91±0.06 |
| DRP-1 / β-tubulin  (Fold change relative to control) | 1.00±0.04 | 2.00±0.10 | 1.32±0.09 |
| FIS1 / β-tubulin  (Fold change relative to control) | 1.00±0.04 | 1.83±0.19 | 2.10±0.25 |

Data represent mean±SEM. The difference between groups was shown in Figures.
